# EPIGENETIC PACE OF AGING (DUNEDINPACE) PREDICTS INCIDENT METABOLIC SYNDROME IN THE BERLIN AGING STUDY II (BASE-II)

**DOI:** 10.1093/geroni/igae098.2623

**Published:** 2024-12-31

**Authors:** Ilja Demuth, Valentin Vetter, Jan Homann, Christina Lill, Lars Bertram

**Affiliations:** Charité - Universitätsmedizin Berlin, Berlin, Berlin, Germany; Charité - Universitätsmedizin Berlin, Berlin, Berlin, Germany; University of Münster, Münster, Nordrhein-Westfalen, Germany; University of Münster, Münster, Nordrhein-Westfalen, Germany; University of Lübeck, Lübeck, Schleswig-Holstein, Germany

## Abstract

The metabolic syndrome (MetS) is highly prevalent in the general population and associated with increased morbidity and mortality. Its diagnosis is based on cut-off values for five cardiovascular risk factors, three of which must be met to make the diagnosis. Epigenetic clocks (Horvath, GrimAge) can be used to quantify differences in biological age as deviation from DNA methylation age (DNAmA) from chronological age, DNAmA acceleration. Another epigenetic biomarker, DunedinPACE, reflects the pace of aging in number of biological years passed per additional chronological year. Aim of the current study is to evaluate the capacity of these different epigenetic markers to predict incident MetS in the BASE-II follow-up dataset assessed on average 7.4 years after baseline. Following exclusion of participants with prevalent MetS a total sample of n= 564 participants (68.3 ± 3.4 years, 55.9% women) with epigenetic biomarkers (baseline) and information on MetS status (follow-up, n=144 incident MetS cases) was available and included in the analyses. Analyses were performed as logistic regressions adjusted for age, sex, time to follow-up, overall morbidity and life style factors. Preliminary analyses revealed that the odds of being diagnosed with MetS after the follow-up period of on average 7.4 years was significantly increased with higher pace of aging assessed as baseline DunedinPACE (OR: 10.7 (95%CI: 1.5-76.1); p = 0.018). Comprehensive results including ROC statistics will be presented at the meeting and discussed in the context of the literature.

